# *catena*-Poly[[bis­(nitrato-κ*O*)cadmium]bis­[μ-1,4-bis­(pyridin-3-yl­meth­oxy)benzene-κ^2^ *N*:*N*′]]

**DOI:** 10.1107/S1600536811032697

**Published:** 2011-08-17

**Authors:** Shuang Zhang, Ying-Hui Yu, Ying Liu, Guang-Feng Hou, Jin-Sheng Gao

**Affiliations:** a Engineering Research Center of Pesticides of Heilongjiang University, Heilongjiang University, Harbin 150050, People’s Republic of China; b College of Chemistry and Materials Science, Heilongjiang University, Harbin 150080, People’s Republic of China

## Abstract

In the title compound, [Cd(NO_3_)_2_(C_18_H_16_N_2_O_2_)_2_]_*n*_, the six-coordinated Cd^II^ ion is located on an inversion center and has a distorted octa­hedral environment defined by four N atoms from four 1,4-bis­(pyridin-3-ylmeth­oxy)benzene ligands and two O atoms from two nitrate anions. The ligands link the Cd^II^ ions into a ribbon-like structure running along [201]. One O atom of the nitrate anion is disordered over two positions with site-occupancy factors of 0.59 (2) and 0.41 (2).

## Related literature

For the synthesis and background to metal complexes with pyridyl-based aromatic ligands, see: Liu *et al.* (2010*a* ▶,*b* ▶). For isotypic compounds, see: Liu *et al.* (2011 ▶); Zou *et al.* (2011 ▶).
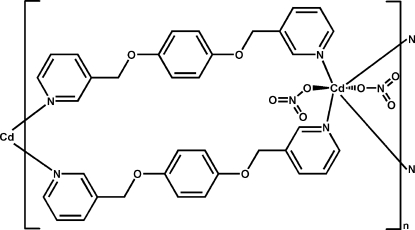


## Experimental

### 

#### Crystal data

[Cd(NO_3_)_2_(C_18_H_16_N_2_O_2_)_2_]
*M* *_r_* = 821.08
Monoclinic, 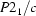

*a* = 8.4034 (17) Å
*b* = 16.914 (3) Å
*c* = 13.436 (5) Å
β = 114.23 (2)°
*V* = 1741.5 (8) Å^3^
*Z* = 2
Mo *K*α radiation
μ = 0.70 mm^−1^
*T* = 293 K
0.20 × 0.19 × 0.17 mm

#### Data collection

Rigaku R-AXIS RAPID diffractometer
Absorption correction: multi-scan (*ABSCOR*; Higashi, 1995 ▶) *T*_min_ = 0.871, *T*_max_ = 0.890
16413 measured reflections
3952 independent reflections
3277 reflections with *I* > 2σ(*I*)
*R*_int_ = 0.028

#### Refinement

*R*[*F* ^2^ > 2σ(*F* ^2^)] = 0.029
*wR*(*F* ^2^) = 0.077
*S* = 1.08
3952 reflections
251 parameters
12 restraints
H-atom parameters constrained
Δρ_max_ = 0.45 e Å^−3^
Δρ_min_ = −0.30 e Å^−3^

### 

Data collection: *RAPID-AUTO* (Rigaku, 1998 ▶); cell refinement: *RAPID-AUTO*; data reduction: *CrystalStructure* (Rigaku/MSC, 2002 ▶); program(s) used to solve structure: *SHELXS97* (Sheldrick, 2008 ▶); program(s) used to refine structure: *SHELXL97* (Sheldrick, 2008 ▶); molecular graphics: *DIAMOND* (Brandenburg, 1999 ▶); software used to prepare material for publication: *SHELXTL* (Sheldrick, 2008 ▶).

## Supplementary Material

Crystal structure: contains datablock(s) I, global. DOI: 10.1107/S1600536811032697/hy2453sup1.cif

Structure factors: contains datablock(s) I. DOI: 10.1107/S1600536811032697/hy2453Isup2.hkl

Additional supplementary materials:  crystallographic information; 3D view; checkCIF report

## Figures and Tables

**Table 1:** Selected bond lengths (Å)

Cd1—N1	2.3793 (17)
Cd1—N2^i^	2.3064 (17)
Cd1—O3	2.3778 (17)

Symmetry code: (i) 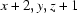.

**Fig. 1.**
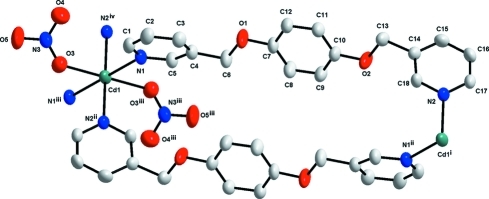
The structure of the title compound, showing displacement ellipsoids at the 50% probability level. H atoms and disordered O5' atom have been omitted for clarity. [Symmetry codes: (i) -2+x, y, -1+z; (ii) -x, 1-y, -z; (iii) 2-x, 1-y, 1-z; (iv) 2+x, y, 1+z.]

**Fig. 2.**
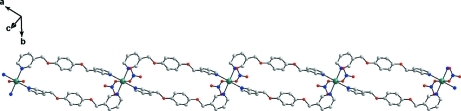
A packing view of the title compound, showing the ribbon-like structure along [2 0 1].

## supplementary crystallographic information

### Comment

The bridging compounds with rigid and flexible pyridyl-containing bidentate or multidentate organic spacers have assembled numerous interesting topology structures by coordination with metals and intermolecular supramolecular interactions. Our group focused attention on study of flexible pyridyl-based aromatic ligands, and obtained some isolated molecules, chain, two- and three-dimensional network structures (Liu *et al.*, 2010*a*, *b*). Herein, as a continuing work for pyridyl ligands, we report the synthesis and crystal structure of the title compound, which is a isostructural compound of our previous reports (Liu *et al.*, 2011; Zou *et al.*, 2011).

In the title compound, the Cd^II^ ion lies on an inversion center and is six-coordinated in a distorted octahedral geometry defined by four N atoms of the pyridine derivatives and two O atoms of the nitrate anions (Fig. 1, Table 1). One O atom of the nitrate anion has a badly thermal ellipsoid, which is split over two positions with site-occupancy factors of 0.59 (2) and 0.41 (2). In the crystal, ribbon-like structures along [2 0 1] are built up by the N-heterocyclic ligands linking the Cd^II^ ions (Fig. 2).

### Experimental

The 1,4-bis(pyridin-3-ylmethoxy)benzene ligand was synthesized as the reference method (Liu *et al.*, 2010*a*). A mixture of 1,4-dihydroxybenzene (1.10 g, 10 mmol), 3-chloromethylpyridine hydrochloride (3.28 g, 20 mmol) and NaOH (1.60 g, 40 mmol) in acetonitrile (50 ml) was refluxed under nitrogen with stirring for 24 h. After cooling to room temperature, the solution was filtered and the residue was washed with acetonitrile for several times. The mixed filtrate was dropped into a 300 ml water solution, giving a powder crude product. A total of 2.51 g (yield 86%) pure product was obtained by recrystallizing from a mixed solution of 10 ml water and 10 ml methanol. The title compound was synthesized by the reaction of 1,4-bis(pyridin-3-ylmethoxy)benzene (0.29 g, 1.0 mmol) and Cd(NO_3_)_2_.4H_2_O (0.31 g, 1.0 mmol) in a mixed solution of 5 ml water and 5 ml methanol. The mixture was filtered after stirring for about 1 h. The filtate was allowed to stand for 4 d under room temperature to give block-like colorless crystals suitable for X-ray analysis.

### Refinement

O5 atom of nitrate is disordered over two positions and the site-occupancy factors were refined to 0.41 (2) for O5 and 0.59 (2) for O5'. The command "isor 0.005 O5 O5" was used to restrain ADP. H atoms bound to C atoms were placed in calculated positions and treated as riding on their parent atoms, with C—H = 0.93 (aromatic) and 0.97 (methylene) Å and with *U*_iso_(H) = 1.2*U*_eq_(C).

### Crystal data

**Table:**

[Cd(NO_3_)_2_(C_18_H_16_N_2_O_2_)_2_]	*F*(000) = 836
*M**_r_* = 821.08	*D*_x_ = 1.566 Mg m^−^^3^
Monoclinic, *P*2_1_/*c*	Mo *K*α radiation, λ = 0.71073 Å
Hall symbol: -P 2ybc	Cell parameters from 14136 reflections
*a* = 8.4034 (17) Å	θ = 3.3–27.5°
*b* = 16.914 (3) Å	µ = 0.70 mm^−^^1^
*c* = 13.436 (5) Å	*T* = 293 K
β = 114.23 (2)°	Block, colorless
*V* = 1741.5 (8) Å^3^	0.20 × 0.19 × 0.17 mm
*Z* = 2	

### Data collection

**Table:**

Rigaku R-AXIS RAPID diffractometer	3952 independent reflections
Radiation source: rotation anode	3277 reflections with *I* > 2σ(*I*)
graphite	*R*_int_ = 0.028
ω scan	θ_max_ = 27.5°, θ_min_ = 3.3°
Absorption correction: multi-scan (*ABSCOR*; Higashi, 1995)	*h* = −8→10
*T*_min_ = 0.871, *T*_max_ = 0.890	*k* = −21→21
16413 measured reflections	*l* = −17→17

### Refinement

**Table:**

Refinement on *F*^2^	Primary atom site location: structure-invariant direct methods
Least-squares matrix: full	Secondary atom site location: difference Fourier map
*R*[*F*^2^ > 2σ(*F*^2^)] = 0.029	Hydrogen site location: inferred from neighbouring sites
*wR*(*F*^2^) = 0.077	H-atom parameters constrained
*S* = 1.08	*w* = 1/[σ^2^(*F*_o_^2^) + (0.0425*P*)^2^ + 0.3288*P*] where *P* = (*F*_o_^2^ + 2*F*_c_^2^)/3
3952 reflections	(Δ/σ)_max_ = 0.001
251 parameters	Δρ_max_ = 0.45 e Å^−^^3^
12 restraints	Δρ_min_ = −0.30 e Å^−^^3^

### Fractional atomic coordinates and isotropic or equivalent isotropic displacement parameters (Å^2^)

**Table:**

	*x*	*y*	*z*	*U*_iso_*/*U*_eq_	Occ. (<1)
Cd1	1.0000	0.5000	0.5000	0.04071 (9)	
O1	0.18190 (19)	0.65049 (9)	0.17002 (17)	0.0667 (5)	
O2	−0.52415 (19)	0.61347 (9)	−0.08007 (16)	0.0643 (5)	
O3	1.2330 (2)	0.56597 (9)	0.47758 (15)	0.0567 (4)	
O4	1.2207 (3)	0.68782 (11)	0.51540 (19)	0.0791 (6)	
O5	1.4627 (9)	0.6264 (6)	0.5848 (14)	0.079 (3)	0.41 (2)
O5'	1.4608 (7)	0.6401 (4)	0.5304 (12)	0.087 (2)	0.59 (2)
N1	0.7748 (2)	0.58067 (10)	0.37477 (14)	0.0411 (4)	
N2	−0.9888 (2)	0.57398 (9)	−0.35307 (13)	0.0370 (3)	
N3	1.3053 (2)	0.62879 (10)	0.51369 (16)	0.0463 (4)	
C1	0.8208 (3)	0.63692 (13)	0.32227 (18)	0.0446 (5)	
H1	0.9391	0.6463	0.3424	0.054*	
C2	0.7023 (3)	0.68165 (14)	0.23999 (18)	0.0469 (5)	
H2	0.7401	0.7213	0.2069	0.056*	
C3	0.5261 (3)	0.66720 (12)	0.20688 (17)	0.0429 (5)	
H3	0.4436	0.6966	0.1509	0.052*	
C4	0.4750 (2)	0.60814 (12)	0.25868 (17)	0.0388 (4)	
C5	0.6032 (2)	0.56757 (12)	0.34290 (17)	0.0418 (4)	
H5	0.5687	0.5291	0.3795	0.050*	
C6	0.2865 (3)	0.58475 (13)	0.2223 (2)	0.0509 (6)	
H6A	0.2595	0.5404	0.1722	0.061*	
H6B	0.2640	0.5691	0.2848	0.061*	
C7	0.0068 (2)	0.63792 (12)	0.10806 (19)	0.0442 (5)	
C8	−0.0774 (3)	0.56579 (13)	0.0975 (2)	0.0517 (6)	
H8	−0.0150	0.5214	0.1336	0.062*	
C9	−0.2549 (3)	0.56010 (13)	0.0329 (2)	0.0507 (6)	
H9	−0.3113	0.5117	0.0251	0.061*	
C10	−0.3483 (2)	0.62632 (12)	−0.01978 (18)	0.0433 (5)	
C11	−0.2652 (2)	0.69830 (11)	−0.00903 (17)	0.0402 (4)	
H11	−0.3282	0.7428	−0.0441	0.048*	
C12	−0.0864 (2)	0.70393 (12)	0.05461 (18)	0.0415 (4)	
H12	−0.0297	0.7521	0.0612	0.050*	
C13	−0.6311 (3)	0.67955 (12)	−0.12538 (19)	0.0476 (5)	
H13A	−0.5876	0.7096	−0.1704	0.057*	
H13B	−0.6339	0.7137	−0.0681	0.057*	
C14	−0.8113 (2)	0.64770 (11)	−0.19354 (16)	0.0376 (4)	
C15	−0.9549 (3)	0.66096 (13)	−0.17100 (19)	0.0472 (5)	
H15	−0.9444	0.6897	−0.1096	0.057*	
C16	−1.1153 (3)	0.63084 (13)	−0.2412 (2)	0.0484 (5)	
H16	−1.2136	0.6390	−0.2272	0.058*	
C17	−1.1282 (2)	0.58893 (11)	−0.33157 (18)	0.0409 (4)	
H17	−1.2369	0.5703	−0.3794	0.049*	
C18	−0.8348 (2)	0.60285 (12)	−0.28455 (16)	0.0385 (4)	
H18	−0.7377	0.5922	−0.2989	0.046*	

### Atomic displacement parameters (Å^2^)

**Table:**

	*U*^11^	*U*^22^	*U*^33^	*U*^12^	*U*^13^	*U*^23^
Cd1	0.02736 (11)	0.04911 (14)	0.03548 (12)	0.00624 (8)	0.00257 (8)	−0.00838 (8)
O1	0.0250 (7)	0.0479 (8)	0.1035 (15)	0.0012 (6)	0.0023 (8)	0.0162 (9)
O2	0.0304 (8)	0.0442 (8)	0.0853 (13)	−0.0014 (6)	−0.0098 (8)	0.0059 (8)
O3	0.0497 (9)	0.0542 (9)	0.0639 (11)	−0.0053 (7)	0.0210 (8)	−0.0034 (8)
O4	0.0698 (12)	0.0553 (10)	0.1048 (17)	0.0078 (9)	0.0284 (12)	−0.0021 (10)
O5	0.036 (3)	0.091 (4)	0.090 (5)	−0.005 (2)	0.005 (3)	−0.006 (3)
O5'	0.0406 (19)	0.095 (3)	0.120 (5)	−0.0096 (17)	0.029 (3)	0.005 (3)
N1	0.0270 (8)	0.0488 (9)	0.0400 (9)	0.0048 (7)	0.0061 (7)	−0.0008 (7)
N2	0.0265 (7)	0.0418 (8)	0.0360 (9)	0.0024 (6)	0.0060 (6)	−0.0032 (7)
N3	0.0334 (9)	0.0447 (10)	0.0595 (12)	0.0035 (8)	0.0179 (8)	0.0089 (8)
C1	0.0283 (9)	0.0557 (12)	0.0463 (12)	−0.0028 (9)	0.0117 (9)	−0.0069 (9)
C2	0.0390 (11)	0.0551 (12)	0.0461 (12)	−0.0068 (9)	0.0170 (9)	0.0028 (9)
C3	0.0364 (10)	0.0489 (11)	0.0354 (10)	0.0022 (9)	0.0065 (8)	0.0043 (8)
C4	0.0269 (9)	0.0439 (10)	0.0389 (11)	0.0026 (8)	0.0066 (8)	0.0017 (8)
C5	0.0280 (9)	0.0469 (11)	0.0442 (11)	0.0037 (8)	0.0083 (8)	0.0063 (9)
C6	0.0262 (9)	0.0529 (12)	0.0623 (15)	0.0023 (9)	0.0066 (9)	0.0142 (10)
C7	0.0232 (9)	0.0484 (11)	0.0541 (13)	0.0008 (8)	0.0087 (9)	0.0058 (9)
C8	0.0315 (10)	0.0429 (11)	0.0677 (15)	0.0043 (9)	0.0071 (10)	0.0130 (10)
C9	0.0322 (10)	0.0402 (10)	0.0659 (15)	−0.0028 (8)	0.0061 (10)	0.0039 (10)
C10	0.0274 (9)	0.0447 (11)	0.0460 (12)	0.0002 (8)	0.0031 (8)	−0.0001 (8)
C11	0.0315 (9)	0.0398 (10)	0.0415 (11)	0.0046 (8)	0.0072 (8)	0.0059 (8)
C12	0.0301 (9)	0.0402 (10)	0.0493 (12)	−0.0015 (8)	0.0115 (9)	0.0046 (8)
C13	0.0339 (10)	0.0446 (11)	0.0478 (12)	0.0030 (9)	0.0000 (9)	−0.0073 (9)
C14	0.0300 (9)	0.0370 (9)	0.0362 (10)	0.0034 (7)	0.0038 (8)	−0.0026 (7)
C15	0.0460 (12)	0.0458 (11)	0.0478 (12)	0.0039 (9)	0.0172 (10)	−0.0121 (9)
C16	0.0339 (10)	0.0489 (11)	0.0660 (15)	0.0039 (9)	0.0241 (10)	−0.0066 (10)
C17	0.0252 (9)	0.0385 (10)	0.0518 (12)	0.0006 (7)	0.0085 (8)	−0.0002 (8)
C18	0.0260 (9)	0.0474 (10)	0.0372 (10)	0.0017 (8)	0.0079 (8)	−0.0048 (8)

### Geometric parameters (Å, °)

**Table:**

Cd1—N1	2.3793 (17)	C5—H5	0.9300
Cd1—N2^i^	2.3064 (17)	C6—H6A	0.9700
Cd1—O3	2.3778 (17)	C6—H6B	0.9700
O1—C7	1.378 (2)	C7—C12	1.383 (3)
O1—C6	1.413 (3)	C7—C8	1.388 (3)
O2—C10	1.380 (2)	C8—C9	1.388 (3)
O2—C13	1.407 (2)	C8—H8	0.9300
O3—N3	1.221 (2)	C9—C10	1.384 (3)
O4—N3	1.231 (3)	C9—H9	0.9300
O5—O5'	0.761 (9)	C10—C11	1.382 (3)
O5—N3	1.275 (7)	C11—C12	1.394 (3)
O5'—N3	1.246 (5)	C11—H11	0.9300
N1—C1	1.333 (3)	C12—H12	0.9300
N1—C5	1.344 (2)	C13—C14	1.511 (3)
N2—C18	1.335 (2)	C13—H13A	0.9700
N2—C17	1.341 (3)	C13—H13B	0.9700
C1—C2	1.372 (3)	C14—C15	1.378 (3)
C1—H1	0.9300	C14—C18	1.383 (3)
C2—C3	1.381 (3)	C15—C16	1.387 (3)
C2—H2	0.9300	C15—H15	0.9300
C3—C4	1.383 (3)	C16—C17	1.371 (3)
C3—H3	0.9300	C16—H16	0.9300
C4—C5	1.382 (3)	C17—H17	0.9300
C4—C6	1.506 (3)	C18—H18	0.9300
			
N2^ii^—Cd1—N2^i^	180.000 (1)	O1—C6—H6A	110.1
N2^ii^—Cd1—O3^iii^	96.17 (6)	C4—C6—H6A	110.1
N2^i^—Cd1—O3^iii^	83.83 (6)	O1—C6—H6B	110.1
N2^ii^—Cd1—O3	83.83 (6)	C4—C6—H6B	110.1
N2^i^—Cd1—O3	96.17 (6)	H6A—C6—H6B	108.4
O3^iii^—Cd1—O3	180.00 (7)	O1—C7—C12	115.21 (18)
N2^ii^—Cd1—N1	87.97 (6)	O1—C7—C8	124.79 (18)
N2^i^—Cd1—N1	92.03 (6)	C12—C7—C8	120.00 (18)
O3^iii^—Cd1—N1	84.33 (6)	C9—C8—C7	119.80 (19)
O3—Cd1—N1	95.67 (6)	C9—C8—H8	120.1
N2^ii^—Cd1—N1^iii^	92.03 (6)	C7—C8—H8	120.1
N2^i^—Cd1—N1^iii^	87.97 (6)	C10—C9—C8	120.14 (19)
O3^iii^—Cd1—N1^iii^	95.67 (6)	C10—C9—H9	119.9
O3—Cd1—N1^iii^	84.33 (6)	C8—C9—H9	119.9
N1—Cd1—N1^iii^	180.0	O2—C10—C11	125.03 (18)
C7—O1—C6	118.12 (16)	O2—C10—C9	114.74 (18)
C10—O2—C13	117.98 (16)	C11—C10—C9	120.22 (18)
N3—O3—Cd1	131.24 (14)	C10—C11—C12	119.73 (18)
C1—N1—C5	117.12 (17)	C10—C11—H11	120.1
C1—N1—Cd1	117.65 (13)	C12—C11—H11	120.1
C5—N1—Cd1	124.68 (14)	C7—C12—C11	120.10 (18)
C18—N2—C17	117.90 (17)	C7—C12—H12	120.0
C18—N2—Cd1^iv^	118.42 (13)	C11—C12—H12	120.0
C17—N2—Cd1^iv^	123.67 (13)	O2—C13—C14	106.41 (16)
O3—N3—O4	121.06 (19)	O2—C13—H13A	110.4
O3—N3—O5'	121.2 (3)	C14—C13—H13A	110.4
O4—N3—O5'	116.3 (3)	O2—C13—H13B	110.4
O3—N3—O5	117.6 (5)	C14—C13—H13B	110.4
O4—N3—O5	116.2 (4)	H13A—C13—H13B	108.6
N1—C1—C2	123.18 (19)	C15—C14—C18	117.61 (18)
N1—C1—H1	118.4	C15—C14—C13	123.97 (19)
C2—C1—H1	118.4	C18—C14—C13	118.41 (19)
C1—C2—C3	119.3 (2)	C14—C15—C16	119.0 (2)
C1—C2—H2	120.3	C14—C15—H15	120.5
C3—C2—H2	120.3	C16—C15—H15	120.5
C2—C3—C4	118.61 (19)	C17—C16—C15	119.6 (2)
C2—C3—H3	120.7	C17—C16—H16	120.2
C4—C3—H3	120.7	C15—C16—H16	120.2
C5—C4—C3	118.17 (18)	N2—C17—C16	121.99 (18)
C5—C4—C6	119.84 (19)	N2—C17—H17	119.0
C3—C4—C6	121.92 (18)	C16—C17—H17	119.0
N1—C5—C4	123.56 (19)	N2—C18—C14	123.82 (18)
N1—C5—H5	118.2	N2—C18—H18	118.1
C4—C5—H5	118.2	C14—C18—H18	118.1
O1—C6—C4	108.15 (17)		

Symmetry codes: (i) *x*+2, *y*, *z*+1; (ii) −*x*, −*y*+1, −*z*; (iii) −*x*+2, −*y*+1, −*z*+1; (iv) *x*−2, *y*, *z*−1.
